# Novel Targeted Therapeutic Strategies for Ewing Sarcoma

**DOI:** 10.3390/cancers14081988

**Published:** 2022-04-14

**Authors:** Daria Fayzullina, Sergey Tsibulnikov, Mikhail Stempen, Brett A. Schroeder, Naveen Kumar, Rajesh Kumar Kharwar, Arbind Acharya, Peter Timashev, Ilya Ulasov

**Affiliations:** 1 Group of Experimental Biotherapy and Diagnostic, Department of Advanced Materials, Institute for Regenerative Medicine, Sechenov First Moscow State Medical University, Moscow 119991, Russia; 2 World-Class Research Center “Digital Biodesign and Personalized Healthcare”, Sechenov First Moscow State Medical University, Moscow 119991, Russia; dfaizullina@yandex.ru (D.F.); ser-tsibulnikov@yandex.ru (S.T.); stempen_m_yu@student.sechenov.ru (M.S.); timashev_p_s@staff.sechenov.ru (P.T.); 3 National Cancer Institute, National Institutes of Health, Bethesda, MD 20814, USA; brett.schroeder@nih.gov; 4 Tumor Immunology Lab, Department of Zoology, Institute of Science, Banaras Hindu University, Varanasi 221005, India; naveentak72@gmail.com (N.K.); acharya@bhu.ac.in (A.A.); 5 Endocrine Research Lab, Department of Zoology, Kutir Post Graduate College, Chakkey, Jaunpur 222146, India; rkkharwar1982@gmail.com; 6 Department of Advanced Materials, Institute for Regenerative Medicine, Sechenov First Moscow State Medical University, Moscow 119991, Russia

**Keywords:** Ewing sarcoma, progression, targeted therapy, *EWSR1/FLI1*

## Abstract

**Simple Summary:**

Ewing sarcoma is an uncommon cancer that arises in mesenchymal tissues and represents the second most widespread malignant bone neoplasm after osteosarcoma in children. Therapy has increased the 5-year survival rate in the last 40 years, although the recurrence rate has remained high. There is an immediate and unmet need for the development of novel Ewing sarcoma therapies. We offer new prospective targets for the therapy of Ewing sarcoma. The EWSR1/FLI1 fusion protein, which is identified in 85–90% of Ewing sarcoma tumors, and its direct targets are given special focus in this study. Experimantal therapy that targets multiple signaling pathways activated during ES progression, alone or in combination with existing regimens, may become the new standard of care for Ewing sarcoma patients, improving patient survival.

**Abstract:**

Ewing sarcoma (ES) is an uncommon cancer that arises in mesenchymal tissues and represents the second most widespread malignant bone neoplasm after osteosarcoma in children. Amplifications in genomic, proteomic, and metabolism are characteristics of sarcoma, and targeting altered cancer cell molecular processes has been proposed as the latest promising strategy to fight cancer. Recent technological advancements have elucidated some of the underlying oncogenic characteristics of Ewing sarcoma. Offering new insights into the physiological basis for this phenomenon, our current review examines the dynamics of ES signaling as it related to both ES and the microenvironment by integrating genomic and proteomic analyses. An extensive survey of the literature was performed to compile the findings. We have also highlighted recent and ongoing studies integrating metabolomics and genomics aimed at better understanding the complex interactions as to how ES adapts to changing biochemical changes within the tumor microenvironment.

## 1. Introduction

Ewing sarcoma (ES) is an aggressive tumor found often in adolescents, accounting for 10% to 15% of all bone sarcomas [1]. The “classic” Ewing bone sarcoma, extra-skeletal ES, malignant small cell tumor of the chest wall (Askin’s tumor), and primitive neuroectodermal tumors based on soft tissue (PNET) were all initially characterized by James Ewing in 1921. The highest incidence occurs in the second decade of life, with approximately 9–10 cases per million per year in patients aged 10–19 compared to an overall incidence of three cases per million per year in the population of the United States [2]. ES occurs predominantly in the Caucasian race and is very rare among African-Americans for unknown reasons [3]. There is a slight predominance of morbidity for men (sex ratio M:F 1.5:1) [3,4]. These tumors have a common genetic background: they are found in the pelvis, but they can appear in any bone or up to 30% of soft tissue [4,5]. Although sarcomas only account for 1% of all human malignancies [6], they are all aggressive [7,8,9] and can rapidly metastasize to the bone marrow, lungs, and other tissues [10,11].

This sarcoma subtype is likely derived from unique progenitor cells with similar genetic backgrounds belonging to endothelial, mesodermal, epithelial, and nerve cells [12,13], but most studies suggest that mesenchymal stem cells (MSCs) are the main precursor cells [14]. Ewing sarcoma belongs to the Ewing sarcoma family tumors (ESFT), which in turn belongs to the FET (FUS-EWSR1-TAF15) group of sarcomas and leukemias. The tumor group comprises more than 20 different tumor entities with ES as one of the most common types [15,16]. These tumors have similar non-random chromosomal translocations with one of the three genes, such as EWSR1, FUS, and TAF15, as 5′ partners and a large group of DNA binding transcription factor encoding genes as 3′ partners. Of note, these fusion proteins encode ETS biding factors that bind to similar DNA sequences. For ES the t(11;22)(q24;q12) translocation is present in 85–90% of tumors and *EWSR1* and *FLI1* gene fusion produces a fusion protein (*EWSR1/FLI1*). The t(11;22) (q24; q12) translocation is found in 85–90% of ES tumors, and the EWSR1 and FLI1 gene fusion results in a fusion protein (EWSR1/FLI1). Further, the t(21;22)(q22;q12), t(7;22)(q22;q12), t(7;22)(q22;q12), t(17;22)(q12;q12), t(2;22)(q33;q12) and others are less common translocations that result in the development of *EWSR1/ERG, EWSR1/ETV1, EWSR1/ETV4* fusions of other genes and are present in the remaining 10–15% of the cases [11,17].

Local treatment and multi-agent adjuvant chemotherapy have increased the 5-year survival rate from less than 20% to more than 70% in the last 40 years, although the recurrence rate has remained high. Approximately 25% of people who have initially confined illness will have it recur at some point in their life. Because there is no standard therapy for recurrent and refractory ES, the 5-year OS for patients with a disease-free interval (DFI) > 2 years is around 30%, and the 5-year OS for those with a DFI of 2 years is about 7%. Given these factors, there is an immediate and unmet need for the development of novel ES therapies [18].

## 2. General Consideration: Hallmarks of Cancer

It is known that cancer cells are characterized by a set of features that distinguish them from non-neoplastic cells. The list of cancer hallmarks has been changed and refined over the years since it was first determined [19]. In 2022, Hanahan edited and expanded the list [20]. Nowadays, the authors of the concept propose nine hallmark capabilities: sustaining proliferative signaling, evading growth suppressors, activating invasion and metastasis, enabling replicative immortality, inducing angiogenesis, resisting cell death, avoiding immune destruction, deregulating cellular energetics, and unlocking phenotypic plasticity. In addition to these, there are also four enabling characteristics, by which cancer cells and tumors can adopt these functional capabilities: genome instability and mutations, tumor-promoting inflammation (with the effect of senescent cells), and non-mutational epigenetic reprogramming, and polymorphic microbiomes.

ES is, in contrast to most other sarcoma types, genetically stable, but the specific chromosomal translocation [11], for example, EWS-FLI1, is necessary for Ewing sarcoma tumorigenicity [21]. As shown earlier by Stolte et al. [21] FET tumors, besides gene fusion, are also characterized by wild type p53, suggesting a dependence of such tumors on DNA damage therapeutic stress, stabilized by p53 signaling. The only protein product of this gene can provide the transformation of cellular processes and the acquisition of hallmarks of cancer. Therefore, our review will focus on several genes and cellular proteins that hold promise for new therapeutic strategies. For ease of discussion and perception, we will consider the changed processes in three clusters: (1) Targeting of ES pressure on adhesion, migration, and invasion, (2) Targeting of Ewing sarcoma cells with a focus on proliferation, cell differentiation, and cell survival, and (3) Targeting of ES: Induction of apoptosis and cell cycle arrest.

## 3. Molecular Targets for ES Therapy

The two most common fusion proteins (*EWSR1/FLI1* and *EWSR1/ERG*) [17] are involved in several cell signaling and regulatory pathways (Graphical Abstract). These fusion proteins usually act as transcription factors. For instance, Boulay et al. [22] identified a chromatin-binding factor (BAF) that interacts with EWSR1/FLI1 to activate gene expression in ES tumor cells and phenotypical changes. On the other hand, oncoprotein fusion can induce target genes via a partnership with GGAA microsatellites, as active enhancers [23] or by binding to RNA. Important discoveries in recent years have shown that major fractions of the ES fusion proteins bind to the SWI/SNF chromatin remodeling complex in tumor cells and that leads to the deregulation of gene expression, such as IGF-1 signaling [24] and epigenetic programming [15,16] towards retaining mesenchymal stem cell plasticity [25]. Blockade of these fusion proteins may be ideal for therapeutic targets. The feasibility of *EWSR1/FLI1* targeting for ES therapy has been shown preclinically using chemical inhibitors [26,27,28] and siRNA technology (Bertrand et al. [29] Gauthier et al. [30] and Cervera et al. [31]) and via chemical targeting of *EWSR1/FLI1* oncogenic fusion with chemical inhibitors [32]. Despite affecting Ewing sarcoma cell viability, multiple reports reveal induction of resistance factors contributing to the tumor’s survival and therapy escape [33,34,35]. Given this problem, it seems prudent to consider the combination of inhibitors against *EWSR1/FLI1* or *EWSR1/ERG* with additional targeted therapies [36,37] that might overcome tumor cells’ resistance to the monotherapy and might demonstrate a less adverse effect due to dose reduction. One such drug is YK-4-279, a drug candidate active in downregulating transcription of EWS/FLI1 [38]. At the same time, YK-4-279 has been shown to inhibit ERG and ETV1 transcription in prostate cancer cells [39], therefore, it might also be active against Ewing sarcoma cells with commonly and less commonly presented fusion in the tumors with Ewing sarcoma. In our review, we want to propose potential targets for therapy that have not yet been used in treatment, along with inhibition of fusion proteins.

### 3.1. Targeting of ES Pressure on Adhesion, Migration, and Invasion

Like most cancer types, the prognosis for ES patients with localized disease is much better than for patients with metastatic disease [40]. A major obstacle in the battle against metastatic disease continues to be an insufficient understanding of underlying processes; specifically, what processes, such as metabolic and others, drive cell adhesion, migration, and invasion during metastases.

Adhesion proteins, such as E-cadherin are regarded as tumor suppressors. The low level of epithelial proteins is associated with spheroid formation and migration [41]. The migration of cancer cells to new niches is a fundamental process underlying metastasis [42]. There are two main types of cancer migration; mesenchymal and amoeboid; each is dependent on complex intracellular signaling that governs the actin cytoskeleton. Actin is involved in several pathways, including Rho/Rac GTPases and the Hippo-pathway, which culminate in the transcriptional regulation of cytoskeletal and growth-promoting genes, respectively. The mesenchymal cell migration of sarcomas can involve both single cells and cells in chains [43]. For successful metastasis, migration is followed by invasion into secondary locations [44].

Cadherins are vital for the formation of cell-cell contacts. During tumor progression, E-cadherin expression is lost, which permits epithelial to mesenchymal transition, anchorage-independent growth, and spheroid formation [45]. Studies have shown that loss of E-cadherin promotes resistance to treatment [40] and acquisition of a mesenchymal-like phenotype in vitro. Ex vivo studies have shown that the increased expression of E-cadherin is associated with improved clinical outcomes in several types of sarcomas [46]. Thus, E-cadherin upregulation in ES cells is mediated by epigenetics [47], or by small molecules like MIL327 [40] or RNA interference to metalloproteinase type 9 (MMP9) [48].

Besides induction of the epithelial to mesenchymal transition (EMT)-developed cell signaling, ES metastasis requires cell migration and blood vessel invasion [49]. The Rho-associated kinases, ROCK1 and ROCK2 have been implemented in the regulation of metastases using various in vitro and in vivo models. Data by Roberto et al. [49] showed a positive correlation between miR-139-5p and ROCK1, where restoration of miR-139-5p impaired ES migration and invasion. Another approach is the application of ROCK2 inhibitors, such as SR3677 and hydroxyfasudil, in SK-ES-1 cells [42] to demonstrate an opportunity for simultaneous targeting of heterogenic ES cells.

Although E-cadherin is generally regarded as an EMT marker, several studies have implicated E-cadherin in tumor adhesion via Ras homolog family member A (Rho A) mediated activation and alterations in paxillin [48]. Although data from Gluer et al. [50] suggested that expression levels of various adhesion markers in ES-based tumor cells matter most, cell adhesion is seemingly determined by the properties of integrin proteins, cadherin 11 [51], and neural cell adhesion molecule (NCAM) [50]. Kang et al. [52] investigated the link between E-cadherin and Erb-B2 Receptor Tyrosine Kinase 4 (ERBB4) using ES spheroids and demonstrated that E-cadherin localizes at cell-to-cell junctions and influences β-catenin cellular localization.

Other cellular proteins, such as integrins and cluster of differentiation 99 (CD99, MIC2) also play roles in ES cell-cell attachment. For example, the affinity of integrin contacts is reduced by CD99, a cell surface protein, via dephosphorylation of focal adhesion kinase (FAK) [53]. Although this protein is not a direct target of *EWSR1/FLI1,* it is important in several signaling pathways [54]. In ES cells, the CD99 protein also impacts cellular adhesion [55], cell growth [56], differentiation [57,58], and tumor cell apoptosis [59] through multiple mechanisms, such as NOTCH-, NFkappa-B, or MAPK modulation. This could be a promising target for therapy since activation with specific monoclonal bodies induces micropinocytosis and leads to cancer cell killing through a caspase-independent, non-apoptotic pathway resembling methuosis [60]. Furthermore, this could be pivotal in tumors with resistance to canonical apoptosis-inducing agents.

The other therapeutic possibility to target ES cells came from understanding the molecular mechanisms that were impacted by the *EWSR1/FLI1* fusion. In sarcoma cells, Katsching et al. [61] reported that *EWSR1/FLI1* directly binds to the proteins belonging to the YAP1/TAZ pathways. Along with another mechanism of Yes-associated protein 1 (YAP)/TAZ regulation, such as AP-1 [62] and activation of Myocardin-related transcription factor B (MRTFB) and TEA domain family member 1 (TEAD) [61], effectors of RhoA and Hippo signaling, *EWSR1/FLI1* has an oscillating mechanism to regulate the balance between the epithelial to mesenchymal transition (EMT), along with the reverse state (MET). These oscillations provide cancer cells with several advantages, including successful invasion, migration, and consequently metastasis. Another transcriptional mechanism contributing to ES metastases involves the Sonic Hedgehog pathway. Previous studies implicated *EWSR1/FLI1* in the regulation of glioma-associated oncogene 1 (GLI1 for humans and GLI1 in mouse models) [63,64,65]. Earlier, micro-array analysis of 27 ES patients showed an association between the Sonic Hedgehog pathway (SHH) and metastasis [66]. Besides metastases, this pathway regulates normal cell growth and proliferation. However, abnormalities can lead to the development of tumors, metastasis, and the emergence of antitumor drug resistance [67]. The binding of an SHH ligand to the patched (Ptch) receptor leads to a cell response expressed in the migration of *GLI1* into the nucleus where it binds to DNA.

The canonical and non-canonical activities of *GLI1* lead to the expression of genes, such as PTCH and SMO, which regulate the cell cycle and proliferation [68]. It has been shown that patient-derived ES cell lines (CHLA9, CHLA10, TC32, CHLA258, and TC71) and tumor samples [69] are enriched with *GLI1*. Abnormalities in this pathway can lead to tumorigenesis, metastasis, and drug resistance [67]. Therefore, YAP / TAZ or SHH pathway blockade holds promise as a drug resistance-preventive strategy. It has been proposed by Joo et al. [65] that the chemical compound GANT58 interferes with shared transcriptional downstream targets between *GLI1* and *EWSR1/FLI1*. GANT61, another synthetic compound derived from hexahydropyrimidine, has been characterized as an inhibitor of GLI1 transcription that binds to the GLI1-DNA complex [70]. It was shown that ES-derived tumor cells can exploit both GLI1 and GLI2 [64], which may minimize the effect of GLI1 inhibitors. Given the use of GANT61 for inhibition of SHH-dependent targets, such as GLI1 [71], additional GLI1 regulation or investigations towards stability offer opportunities to target cellular proteins vital for ES transformation.

Insulin-like growth factor (IGF-1) is associated with several oncogenic processes in cells [36] and the IGF-1 receptor (IGF-1R) is a pivotal receptor tyrosine kinase that regulates malignant tumor transformation of ES cells [72]. IGF-1 and IGF-1R are overexpressed in the majority of Ewing sarcoma cell lines and have been previously studied as potential therapeutic targets in tumor treatment. It was reported that the IGF-1R pathway is activated in several cancers, such as hepatocellular carcinoma [73], pancreatic ductal carcinoma [74], retinoblastoma [75], colorectal cancer [76], and Ewing sarcoma [77,78]. Chromosomal translocation of *EWSR1/FLI1* leads to activation of the IGF-1R pathway and its downstream targets belonging to the Mitogen-Activated Protein Kinase (MAPK) and the Phosphoinositide 3-kinase (PI3K) pathways [79] to maintain the phenotype and viability of ES cells. Although the use of IGF-1R inhibitors was efficacious in vitro, no sustained clinical response was found for ES patients [80].

It was discovered that inhibition of the IGF pathway initiated aberrant compensatory mechanisms, such as pregnancy-associated plasma protein-A (PAPP-A) [81]. Research findings have shown that synergistic action of PAPP-A and IGF-1R was more effective in EWS [81] and hence, their combined treatment could play a potential role in EWS therapy. However, this study was preclinical, and lacked an immunocompetent EWS model. Further research using an EWS model will be helpful for a better understanding of the immunomodulating effects of anti-PAPP-A. PAPP-A is a zinc metalloproteinase that cleaves inhibitory IGF-1-binding proteins, thereby increasing IGF-1 availability for IGF receptor-mediated cell proliferation, migration, and survival. PPAP-A enhances local IGF-1, which is also associated with invasion and metastasis. Potential targetable cell antigens in ES include PAPP-A as one of the top five secreted metalloproteinase proteins overexpressed in ES [81]. Furthermore, it has been shown that therapy with transgenic T cells directed against PAPP-A, knockout of the PAPP-A gene, and complex therapy in which not only PAPP-A is directly inhibited, can also be effective [82].

The TGF-β and PDGF pathways play important roles in ES plasticity and tumor progression. The TGF-β co-receptor endoglin is routinely expressed by malignant cells, and it is associated with the upregulation of bone morphogenetic protein, integrin, focal adhesion kinase, and phosphoinositide-3-kinase signaling, which together work in concert to maintain tumor cell plasticity [43,83]. Like TGF-β, the platelet-derived growth factor (PDGF) pathway aids in maintaining a cancer stem cell-like phenotype in ES, but more notably, is involved in ES tumor neovascularization [84]. Importantly PDGF ligands and/or receptors are frequently upregulated in ES [66,85] and their expression correlates with the activity of the *EWSR1/FLI1* fusion [86] in sarcoma tissues. Overall, the pathways involved in ES cellular invasion and migration are complex, and future work is needed to design an effective multitargeted approach against ES tumor cells [87].

These genes play a key role in several tumor processes for ES, especially in adhesion, migration, and invasion (Table 1). The regulation of these processes in a tumor is the first step toward the formation of a lesion secondary tumor growth-metastasis from a localized tumor. Metastasis is associated with poor clinical outcomes for patients, while the treatment of localized tumors currently has a relatively high success rate, it could be as high as 70% [18]. Therefore, these genes as targets for therapy are especially vital.

### 3.2. Targeting of Ewing Sarcoma Cells with a Focus on Proliferation, Cell Differentiation, and Cell Survival

Metastatic progression requires the migration and invasion of cancer cells to reach distant tissues. However, for malignant neoplasms to spread beyond their primary tumor, they must adapt and thrive in a new niche. In general, cancer cells are characterized by unlimited time spent on cell division coupled with high survivability [88]. The mechanism for preserving these cells in a poorly differentiated state is elaborate. Mutations and epigenetic changes trigger unregulated mitotic cycles, allowing cells to become insensitive to growth-inhibitory signals, and capable of evading programmed cell death. Cell cycle progression and cell division, cell death, and cellular senescence determine cell proliferation in a broad sense.

The expression of the ES fusion gene alters the expression of over 500 downstream targets, which collectively block differentiation and drive proliferation [89]. For example, *EWSR1/FLI1* regulates several genes, including IGF-1, Homeobox protein Nkx (NKX2), T-LAK cell-originated protein kinase (TOPK), SRY-Box Transcription Factor 2 (SOX2), and Enhancer Of ZesteHomolog2 (EZH2) [90]. It is worth noting that EWS-FLI1 binds to the *EZH2* promoter to activate embryonic tumor stem cell growth and metastatic spread [91]. These targets can be used in the treatment of Ewing sarcoma. For example, GSK126 inhibits *EZH2* methyltransferase activity in ES cells. Firstly, it reduces the phenotypic heterogeneity of ES cells and their capability for self-renewal and tumorigenicity. In turn, the change in the concentration of *EZH2* targets (non-protein neuroectodermal marker G_D2_) allows chimeric antigen receptor gene-modified T cell therapy to be applied to the tumor [92]. Ahmed et al. [93] assessed the biomarkers responsible for ES cell proliferation. Immunostaining of primary tissue revealed that the majority were positive for protein kinase B (AKT) (55%) and mTOR (77%), indicating activation of an AKT-mTOR axis in ES cells. Thus, mTOR could be a budding target, some clinical trials for it are in process (see Section 4). Approximately 33% of specimens also expressed YAP, establishing a link between proliferation and YAP. Additionally, YAP is critically associated with BMI-1 (a polycomb complex protein) which stabilizes YAP expression and activity [94] via controlled chromatin remodeling.

Cell survival is connected with another hallmark of cancer deregulating cellular energetics. Numerous metabolic, i.e., catabolic and anabolic processes are altered in ES cells. For instance, Tanner et al. [95] reported altered de novo serine synthesis and dependence on aerobic glycolysis increased instead of oxidative metabolism. Moreover, data from an investigation by Sen et al. [96] demonstrates a direct link between EWSR1/FLI1 proteins involved in serine biosynthesis and glutamine consumption. Interestingly, Issaq et al. [97] showed that the expression of 3-phosphoglycerate dehydrogenase (PHGDH) was regulated by *EWSR1/FLI1*. PHGDH is one of the main enzymes that catalyze3-phosphoglycerate to 3-phosphohydroxypyruvate [98] (serine de novo synthesis) that is required for cell proliferation and tumor growth [97]. Further, PHGDH knockdown decreased ES cell proliferation and inhibited xenograft tumorigenesis in orthotopic ES models, providing an additional link between *EWSR1/FLI1* and ES carcinogenesis. Therefore, targeting serine metabolism could be important for novel anticancer approaches in ES.

It is well-known that several cancers [99,100] heavily rely upon glycolysis rather than oxidative metabolism since it provides metabolic plasticity to fuel tumor heterogeneity [101,102]. Ewing sarcoma is one tumor type [103] where a predominant fusion protein, *EWS/FLI1*, regulates glucose consumption as well as gene expression of glycolytic enzymes, such as lactate dehydrogenase (LDH) [32]. It has been shown recently that depletion of lactate dehydrogenase-A (LDHA) inhibits proliferation of ES cells and induces apoptosis, impacting tumor cell viability both in vitro and in vivo.

Additionally, for various cancers, a growing number of important regulators are being discovered within one group of molecules: long non-coding RNAs (lncRNAs) [104]. LncRNAs often increase cancer cell survival, proliferation, colony formation, migration, and invasion [104,105,106]. Their elevated expression contributes to the progression of the sarcoma. It was described, for example, for lncRNA taurine upregulated gene 1 (TUG1) [106] and lncRNA SOX2 [105]. Many of the biological regulatory mechanisms of lncRNAs in Ewing sarcoma are still elusive. Nevertheless, they have been shown to often act as competing endogenous RNAs to regulate other genes expression. In principle, the knockdown of lncRNAs or the selection of inhibitory proteins might help to suppress ES growth [105,106]. These approaches might be potential therapeutic options for treating Ewing sarcoma.

**Figure 1:**
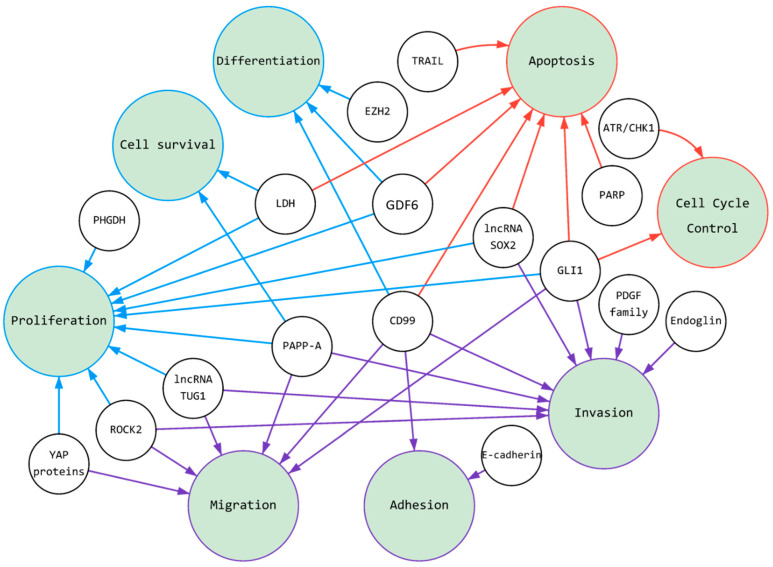
**Schematic illustration of cancer pathways under control of ES’s fusion proteins:** The figure summarizes our review of research active molecules over the past 10 years. The various domain of ES-produced fusion oncoproteins required for the activation of gene expression, and their products, such as RNA or proteins regulate cellular proliferation, apoptosis and migration, for example. All together with induced cell signaling cells gain oncogenic traits and transformation. Small circles are active molecules, large circles are cellular processes (see the hallmark of cancer). Colors in the figure are correlated to organizational divisions in the review for ease of perception. There are three colored clusters: purple (Section 3.1. Targeting of ES Pressure on Adhesion, Migration, and Invasion), blue (Section 3.2; Targeting of Ewing Sarcoma Cells with a Focus on Proliferation, Cell Differentiation, and Cell Survival) and red (Section 3.3; Targeting of ES: Induction of Apoptosis and Cell Cycle Arrest).

In summary, cell proliferation, differentiation, and survival are important, first of all, in the formation of a tumor in a primary or secondary lesion. These properties of cells are related to some extent to other tumor characteristics (Figure 1), for example, deregulated cellular energetics (Table 1). To regulate this group of genes and their products, *EWSR1/FLI1* usually acts as a transcription factor and binds with RNA.

### 3.3. Targeting of ES: Induction of Apoptosis and Cell Cycle Arrest

The development of fusion event inhibitors to suppress the progression of Ewing sarcoma requires the understanding of multiple cellular processes which became active in the tumor cells. Since kinases are integral to tumor maintenance, intervention directed toward these family members is promising. Both apoptosis and cell division utilize several different kinases, such as ATR and CHK1.

ES cells exhibit increased levels of endogenous DNA replicative stress and are sensitive to inhibitors of ribonucleotide reductase (RNR), an enzyme that limits the rate of deoxyribonucleotide synthesis. ES cells are also dependent on the ataxia telangiectasia, the rad3-related protein (ATR), and the checkpoint kinase 1 (CHK1) pathway, which play key roles in orchestrating the cellular response to DNA replication stress for survival [107,108]. ES tumors are sensitive both in vitro and in vivo to ATR and CHK1 inhibitors as separate agents and in combination with other drugs [107,108,109,110,111]. The ATR-CHK1 pathway, when activated by DNA replication stress, orchestrates a multifaceted response that arrests cell cycle progression, suppresses the origin of replication, stabilizes replication forks, and promotes fork repair and restart [112].

However, ATR and CHK1 also have critical and unique functions outside of the S phase and the response to DNA replication stress. For example, ATR and/or CHK1 regulate chromosome segregation, the S/G2 checkpoint, the G2/M transition, double-strand DNA break repair, and the response to osmotic and mechanical stress [113,114]. Recently, Koppenhafer et al. [87] identified that the inhibition of the ATR-CHK1 pathway in ES cells under DNA replication stress leads to the aberrant activation of Cyclin-Dependent Kinase 2 (CDK2) and cell death. CDK1 and CDK2 are critical mediators of cell cycle progression that are regulated by the ATR-CHK1-CDC25A pathway. In the setting of DNA replication stress, ATR-CHK1 negatively regulates CDC25A, which de-phosphorylates and activates CDK1/2 to restrain cell cycle progression and promote DNA damage repair. A novel feedback intracellular loop in Ewing sarcoma cells has been discovered. In this loop, the inhibition of the ATR-CHK1 pathway, or the WEE1 kinase, during DNA replication stress leads to enhanced DNA replication stress, increased DNA damage, and apoptosis. Although most investigations in this field focus on the interaction between *EWS*/*FLI1* and intracellular pathways, several others have explored the possibility of interfering with extracellular signaling paths that regulate *EWS/FLI1*, and consequently, tumor transformation.

Additionally, one of the enzymes regulating the work of the hereditary apparatus of the cell is Poly (ADP-ribose) polymerase (PARP), which is involved in the processes of DNA repair, maintaining the genetic stability of the cell and its programmed death, and as Ewing sarcoma cell lines are frequently defective in DNA break repair, they are susceptible to PARP inhibition [115,116,117]. Inhibition of PARP showed the effectiveness of this approach if cytostatic drugs were used, the effect of which was intensified [118]. Some studies indicate an increase in the effectiveness of treatment of Ewing sarcoma with PARP inhibitors alone [119] or with a combination, such as temozolomide [120,121]. Although preclinical in vitro models showed an acceptable result, the activity of PARP inhibitors as a single agent in preclinical in vivo models and clinical trials at an early stage of Ewing sarcoma did not demonstrate significant results [122]. The activity of PARP directly depends on its required substrate, nicotinamide adenine dinucleotide (NAD+), which is produced by nicotinamide phosphoribosyltransferase (NAMPT). Studies of the combined use of PARP and NAMPT inhibitors in vivo have shown great effectiveness. The combined therapy resulted in tumor regression, delayed disease progression, and increased survival [123]. Considering that Ewing sarcoma cells depend on functioning PARP, that PARP requires NAD+, and that NAD+ production depends on NAMPT, it seems appropriate to simultaneously inhibit these proteins, which are confirmed by recent studies [123].

Growth and differentiation factor 6 (GDF6), also known as bone morphogenetic protein 13 (BMP13), belongs to the TGF-superfamily’s BMP family. GDF6 has become an attractive target since its binding to its own receptor, such as CD99 [41]. GDF6 is vital to embryogenesis, particularly to the development of the neural and skeletal systems, and mutations in GDF6 are associated with abnormalities of the skeleton, the eyes [124], and other organs [125]. GDF6 is highly expressed in ES tumors and cell lines compared to mesenchymal stem cells and cells from other sarcoma subtypes.

Zhou et al. [41] described a GDF6 prodomain signaling pathway that regulates Src activity and ES tumor growth [41] via p21. A ChIP-sequencing showed binding of *EWSR1/FLI1* to the GDF6 gene in ES cells, which implicates GDF6 as a direct target of *EWSR1/FLI1* transcription activation. Considering the role of GDF6 in cell proliferation and differentiation, inactivation of gene expression [41] will offer a possibility to interfere with ES cell growth.

The anticancer properties of multiple ES-based therapeutic approaches have been extensively studied, often for their antiproliferative effects. The induction of apoptosis has been reported for several cancers, including Ewing sarcoma. However, ES cells are prone to developing treatment resistance, which contributes to disease recurrence [126]. Current ES-therapeutic strategies are promising, and the development of new therapeutics and combinations thereof with greater antitumoral properties has been proposed. For instance, Sonnemann et al. [127] investigated cell death mediated by histone deacetylase (HDAC) inhibitors in the presence of pro-apoptotic TNF-related apoptosis-inducing ligand (TRAIL). Additionally, Lu et al. [128] found that the proteasome inhibitor bortezomib synergizes with TRAIL in vitro using TC-71, to enhance cancer cell-related toxicity through apoptosis.

Previously, TRAIL, a member of the tumor necrosis factor (TNF) ligand superfamily (TNFLSF), has been found to induce apoptosis in cancer cells while sparing normal cells [129,130,131]. Multiple in vitro studies have shown that TRAIL and other death receptor agonists [132,133] are effective against sarcoma cell lines [131,134] with ES cell lines showing the greatest sensitivity [135]. Ubiquitin-specific protease 6 (USP6) may also contribute to the sensitization of ES cells to exogenous IFNs [136]. Henrich and colleagues [136] speculate that this negative feedback loop involves USP6, which serves to amplify Interferon Gamma (IFN)-mediated sensitivity to TRAIL. Indisputably, the molecular mechanism of TRAIL sensitivity warrants additional investigation to clarify the molecular basis for drug synergy against ES.

The metabolism of Ewing sarcoma involves many genes and metabolic pathways that may be potential targets for therapy (Table 1). The effect on some agents can lead to a static effect, and the activation or silencing of others can be expressed as a bright antitumor effect and lead to apoptosis. Some experimental approaches to stimulating cell cycle arrest [137,138,139] or apoptosis [140,141] in Ewing sarcoma cells showed effectiveness in preclinical settings, and therefore, might hold great promises in future clinical testing.

## 4. Current Clinical Trials

Standard chemotherapies, such as alkylating agents, topoisomerase, and tubulin inhibitors are non-specific and exert their effects on both tumor cells and normal cells. Currently, a search is underway to identify new drugs that are specifically targeted for ES cells and that are capable of eliminating the tumor cells and extending patient survival. Some clinical trials where traditional and experimental components of chemotherapy were used both separately and in combination with each other are presented (Table 2). The key to new and successful therapies may be the addition of standard treatment protocols with new, highly specific experimental drugs.

## 5. Conclusions

Ewing sarcoma is a cancer with metabolic processes and related pathology largely governed by fusion proteins. Unfortunately, inhibition of these proteins themselves has proved challenging and clinically unsuccessful, which necessitates combinations of new therapeutic approaches. After an extensive literature review, we chose 17 molecules that serve as promising targets for therapy that alter cell metabolism, and possess features crucial in tumorigenesis, including cell adhesion, migration, invasion, proliferation, differentiation, survival, apoptosis, and cell cycle arrest (Figure 1). Most of the genes we have described are direct targets of fusion proteins, therefore, successful indirect inhibition could have a cascading effect on cell survival and might have future clinical implications. In addition, many of the presented proteins are often highly expressed (some serve as markers for ES), so their inhibition could be readily available and exert a strong anti-cancer effect. Clinical trials are currently underway for some of these aforementioned target molecules. Therapies devoted to targeting them alone or in combination with current regimens could become the next standard of care for Ewing sarcoma patients.

## Figures and Tables

**Table 1:** Targeting molecules in ES pathogenesis.

Targetable Molecules	Main Pathways	Tumor Effects
CD99	IGF-1R and RAS-Rac1 signaling	Induces caspase-independent cell death, endocytosis, cell aggregation, micropinocytosis, cell adhesion, migration, invasion, metastasis, differentiation
GDF6	GDF6 prodomain signaling pathway	Cell proliferation, tumor growth, differentiation, apoptosis
E-cadherin	MAPK Pathway	Anchorage-independent growth and spheroid formation, cell-cell adhesion
Endoglin	TGFβ signaling	Tumor cell plasticity, patient survival, invasion, anchorage-independent growth, progression of aggressive tumors
EZH2	Epigenetic	Cell differentiation, phenotypic heterogeneity, self-renewal
GLI1	Sonic Hedgehog (SHH) pathway	Cell proliferation, cell cycle control, apoptosis, cell viability, metastasis, invasion, migration, clonogenicity
PDGF family members	PDGF pathway	Self-renewal, invasion, chemotherapy resistance, primary tumor growth, metastasis, drug resistance, poor clinical outcome
ROCK2	RhoA-ROCK pathway	Migration, invasion, proliferation, clonogenic capacity, tumor growth
YAP proteins	YAP/TAZ pathway, Hippo signaling, WNT/β-catenin signaling	Migration, cell proliferation, metastasis, anchorage-independent colony formation
PAPP-A	IGF signaling	Cell proliferation, migration, cell survival, tumor growth, invasion, metastasis
PARP family	DNA repair, replication	Apoptosis
TRAIL	TRAIL-pathway	Induces caspase-independent cell death, apoptosis
ATR/CHK1	ATR-CHK1 pathway	Cell cycle regulation, cell cycle arrest
LDH	aerobic glycolysis	Cell proliferation, apoptosis, tumor growth, cell survival
PHGDH	Serine synthesis	Cell proliferation
lncRNA SOX2	WNT/β-catenin signaling	Cell proliferation, invasion, apoptosis, tumor growth
lncRNA TUG1	TUG-miR-145-5p-TRPC6 pathway	Cell proliferation, migration, invasion

**Table 2:** Clinical trials.

S.N.	Number	Number of Patients	Disease	Drug/Target	Results
1	NCT04129151	18	Ewing Sarcoma Recurrent	Palbociclib/CDK4 and CDK6 Ganitumab/IGF-1R	Active
2	NCT02546544	16	Relapsed Ewing Sarcoma Refractory Ewing Sarcoma	Linsitinib/IGF-1R	Disease progression, limited therapeutical effect
3	NCT00949325	24	Soft Tissue and Bone Sarcoma	Temsirolimus/mTOR Doxorubicin/topoisomerase II	The response rate was 53%, found a correlation between inhibition of mTOR and therapeutical effect 10.1186/s13569-018-0107-9
4	NCT00987636	907	Ewing sarcoma	Zoledronic acid/osteoclast apoptosis Busulfan/guanine N7 Treosulfan/guanine N7 Melphalan/guanine N7	BuMel treatment was more successful than standard chemotherapy -vincristine, dactinomycin, and ifosfamide (VAI)
5	NCT00618813	35	Ewing Sarcoma	Radiation therapy therapeutic conventional surgery etoposide/topoisomerase II ifosfamide/DNA doxorubicin hydrochloride/topoisomerase II cyclophosphamide/guanine N7 vincristine sulfate/tubulin topotecan hydrochloride/topoisomerase I filgrastim/Granulocyte	No incidence of death was recorded in 37 weeks of treatment
6	NCT00516295	7	Ewing Sarcoma of Bone Extraosseous Ewing Sarcoma Peripheral Primitive Neuroectodermal Tumor Recurrent Ewing Sarcoma/Peripheral Primitive Neuroectodermal Tumor	Topotecan hydrochloride/topoisomerase I cyclophosphamide/guanine N7 vincristine sulfate/tubulin bevacizumab/VEGF-A	Days of event free survival—442
7	NCT00470275	10	Recurrent or Refractory Ewing Sarcoma	Cytarabine/DNA	Lack of efficacy
8	NCT02657005	45	Relapsed or Refractory Ewing Sarcoma	TK216/EWS-FLI1	Active
9	NCT00061893	38	Ewing Sarcoma Family of Tumors	Radiation therapy conventional surgery etoposide/topoisomerase II ifosfamide/DNA doxorubicin hydrochloride/topoisomerase II cyclophosphamide/guanine N7 vincristine sulfate/tubulin topotecan hydrochloride/topoisomerase I filgrastim/granulocyte vinblastine sulfate/tubulin MESNA/urotoxic metabolites	24-month event free survival was 35%: 71% for the seven with isolated pulmonary metastases, 26% for all others.
10	NCT02511132	22	Ewing Sarcoma	Vigil/TGF-β Temozolomide/guanine Irinotecan/topoisomerase I	1 case report of complete response to therapy
11	NCT01583543	12	Recurrent/Metastatic Ewing’s Sarcoma	Olaparib/PARP	No significant responses or durable disease control was seen
12	NCT01331135	18	Ewing sarcoma, osteosarcoma, malignant peripheral nerve sheath tumor, rhabdoid tumor, retinoblastoma	Sirolimus/mTOR	The combination of sirolimus with metronomic chemotherapy is well tolerated in children. A phase II trial of this combination is ongoing.
13	NCT00428272	24	Ewing Sarcoma Osteosarcoma Neuroblastoma Rhabdomyosarcoma	Lexatumumab/TRAIL-2R	The drug seems to mediate some clinical activity in pediatric solid tumors and may work with radiation to enhance antitumor effects.
14	NCT02306161	312	Metastatic Ewing Sarcoma Metastatic Malignant Neoplasm in the Bone Metastatic Malignant Neoplasm in the Bone Marrow Metastatic Malignant Neoplasm in the Lung Metastatic Peripheral Primitive Neuroectodermal Tumor of Bone Peripheral Primitive Neuroectodermal Tumor of Soft Tissues	Cyclophosphamide/guanine N7 Doxorubicin/topoisomerase II Etoposide/topoisomerase II Ganitumab/IGevent-freeF-1R Ifosfamide/DNA Vincristine/tubulin	Active
15	NCT04067115	45	Ewing Sarcoma	Trabectedin/guanine N2 Irinotecan/topoisomerase I	Recruiting
16	NCT00070109	50	Rhabdomyosarcoma Recurrent Childhood Rhabdomyosarcoma Recurrent Childhood Soft Tissue Sarcoma Recurrent Ewing Sarcoma Peripheral Primitive Neuroectodermal Tumor	Trabectedin/guanine N2	
17	NCT03600649	50	Ewing Sarcoma Myxoid Liposarcoma Sarcoma, Soft Tissue Desmoplastic Small Round Cell Tumor Extraskeletal Myxoid Chondrosarcoma Angiomatoid Fibrous Histiocytoma Clear Cell Sarcoma Primary Pulmonary Myxoid Sarcoma Myoepithelial Tumor Sclerosing Epithelioid Fibrosarcoma Fibromyxoid Tumor	Cyclophosphamide/guanine N7 Topotecan/topoisomerase I Seclidemstat/LSD1	Recruiting
18	NCT03491371	56	Osteosarcoma Ewing sarcoma Chondrosarcoma Soft tissue sarcoma	Methylsulfonic apatinib/VEGFR-2	No data
19	NCT04690725	29	Osteosarcoma Ewing sarcoma Chondrosarcoma	TQB3525/PI 3-kinases	Active
20	NCT01610570	8	Ewing Sarcoma Sarcoma	Mithramycin/EWS-FLI1	The trial was closed to enrollment, due to inability to safely achieve the desired mithramycin exposure

## Abbreviation

CD99	cluster of differentiation 99
EMT	epithelial to mesenchymal transition
ERBB4	Erb-B2 Receptor Tyrosine Kinase 4
ES	Ewing sarcoma
ESFT	Ewing sarcoma family tumors
EZH2	Enhancer Of Zeste 2 Polycomb Repressive Complex 2 Subunit
GDF6	growth and differentiation factor 6
FET	family of genes, Fused in sarcoma, Ewing sarcoma breakpoint region 1, TATA-box binding protein associated factor 15
GLI1	glioma-associated oncogene 1
IGF-1	Insulin-like growth factor
IGF-1R	insulin-like growth factor 1 receptor
LDH	lactate dehydrogenase
lncRNAs	long non-coding RNAs
MAPK	Mitogen-Activated Protein Kinase
MET	mesenchymal to epithelial transition
MMP9	metalloproteinase type 9
MRTFB	of Myocardin-related transcription factor B
NAMPT	nicotinamide phosphoribosyl transferase
NCAM	neural cell adhesion molecule
PAPP-A	Pregnancy-associated plasma protein A
PARP	Poly (ADP-ribose) polymerase
PDGF	Platelet-derived growth factor
PHGDH	3-phosphoglycerate dehydrogenase
PI3K	Phosphoinositide 3-kinases
ROCK	Rho-associated coiled-coil kinase
RhoA	Ras homolog family member A
SHH	Sonic Hedgehog pathway
SOX2	SRY-Box Transcription Factor 2
SRBCT	Small Round Blue Cell Tumors
TEAD	TEA domain family member 1
TNF	tumor necrosis factor
mTOR	mammalian target of rapamycin
TRAIL	TNF-Related Apoptosis Inducing Ligand
PNET	Primitive neuroectodermal tumor
VEGF-A	Vascular endothelial growth factor A
YAP	Yes-associated protein.
